# Inhibition of serum and glucocorticoid regulated kinase-1 as novel therapy for cardiac arrhythmia disorders

**DOI:** 10.1038/s41598-017-00413-3

**Published:** 2017-03-23

**Authors:** Vassilios J. Bezzerides, Aifeng Zhang, Ling Xiao, Bridget Simonson, Santosh A. Khedkar, Shiro Baba, Filomena Ottaviano, Stacey Lynch, Katherine Hessler, Alan C. Rigby, David Milan, Saumya Das, Anthony Rosenzweig

**Affiliations:** 1 Beth Israel Deaconess Medical Center, Boston, MA USA; 2 Boston Children’s Hospital, Department of Cardiology, Boston, MA USA; 3 Warp Drive Bio Inc., Cambridge, MA USA; 4 Massachusetts General Hospital, Boston, MA USA; 5 Graduate School of Medicine Kyoto University, Kyoto City, Japan; 6 ChemBio Discovery Solutions, Lexington, MA USA

## Abstract

Alterations in sodium flux (I_Na_) play an important role in the pathogenesis of cardiac arrhythmias and may also contribute to the development of cardiomyopathies. We have recently demonstrated a critical role for the regulation of the voltage-gated sodium channel Na_V_1.5 in the heart by the serum and glucocorticoid regulated kinase-1 (SGK1). Activation of SGK1 in the heart causes a marked increase in both the peak and late sodium currents leading to prolongation of the action potential duration and an increased propensity to arrhythmia. Here we show that SGK1 directly regulates Na_V_1.5 channel function, and genetic inhibition of SGK1 in a zebrafish model of inherited long QT syndrome rescues the long QT phenotype. Using computer-aided drug discovery coupled with *in vitro* kinase assays, we identified a novel class of SGK1 inhibitors. Our lead SGK1 inhibitor (5377051) selectively inhibits SGK1 in cultured cardiomyocytes, and inhibits phosphorylation of an SGK1-specific target as well as proliferation in the prostate cancer cell line, LNCaP. Finally, 5377051 can reverse SGK1’s effects on Na_V_1.5 and shorten the action potential duration in induced pluripotent stem cell (iPSC)-derived cardiomyocytes from a patient with a gain-of-function mutation in Nav 1.5 (Long QT3 syndrome). Our data suggests that SGK1 inhibitors warrant further investigation in the treatment of cardiac arrhythmias.

## Introduction

Sudden cardiac death (SCD) is a leading contributor to mortality in the United States. With a current incidence of 180,000 to 450,000 per year^1^, the rate of SCD is likely to increase with the aging of the population. Disorders of sodium flux (I_Na_) have recently been shown to play an important role in the pathogenesis of cardiac arrhythmias in both acquired and inherited arrhythmia syndromes^2^. Notably, mutations in the *SCN5a* gene that encodes the primary cardiac voltage-gated sodium channel, Na_V_1.5, cause multiple inherited arrhythmia syndromes, including long QT syndrome 3 (LQT3), atrial fibrillation, and conduction disorders^3^. Interestingly, alterations in I_Na_ have also been observed in heart failure (HF), and may contribute to HF progression^4, 5^.

Medications that reverse abnormalities in I_Na_ may therefore have a role in the treatment of arrhythmias associated with primary inherited channelopathies, as well as acquired heart diseases. Despite some success in using anti-arrhythmic drugs to modulate SCD risk in either animal or small human studies^6, 7^, attempts to target I_Na_ have not effectively been translated to the wider population, in part due to the pro-arrhythmic and negative inotropic side effects of many anti-arrhythmic agents. Consequently, treatment of SCD in high risk patients relies on the implantation of internal cardiac defibrillators (ICDs), an invasive procedure associated with significant cost and potential morbidity^8^. Therefore, there is a clear unmet need for novel therapeutic approaches to the treatment of arrhythmias in these disease populations.

We have recently identified the serum and glucocorticoid-regulated kinase-1 (SGK1) as an important regulator of I_Na_ in the heart^4^. Of the three known isoforms, SGK1 and SGK3 are predominately expressed in the heart^9^. Unlike the related kinase Akt1, SGK1 is an important regulator of Na^+^ and K^+^ channels^4, 10, 11^. Prior studies have implicated SGK1 in the regulation of the epithelial Na^+^ channel, voltage-gated K^+^ and Na^+^ channels, and other ionic transporters mostly by regulating their trafficking to the cell membrane^12–15^. Notably, SGK1 appears to be activated predominantly in pathological conditions^16^, and inhibition of SGK1 either by germ-line ablation^17^ or by dominant-negative genetic inhibition does not cause a noticeable phenotype under basal conditions, but appears to be protective against pathological stress^4^. In contrast, chronic SGK1 activation in cardiomyocytes leads to a marked alteration in the sodium flux (but not of K^+^ or Ca^2+^ fluxes), prolongation of action potential duration (APD) in cardiomyocytes (CMs) and a markedly increased propensity for lethal ventricular arrhythmias^4^. These studies raise the possibility that small molecule inhibitors would be anti-arrhythmic in cardiac diseases through correction of abnormal I_Na_ while having little or no adverse consequences in normal hearts.

Over the past two decades, significant advances in targeted therapies have greatly changed the natural history of patient outcomes in many different cancers^18^. Because of increased survival there has been a growing recognition that many traditional and advanced chemotherapeutics can adversely affect the cardiovascular system resulting in significant clinical complications^19^. More recently, the specific inhibition of key protein kinases has ushered in a new era of targeted therapy for oncology as dysregulation of protein signaling pathways are often associated with cancer progression. However, these therapies also have specific cardiac toxicities because many of signaling pathways targeted in cancer also are important in cardiac function and growth^20^. There now exists an enhanced awareness of the negative effects of chemotherapeutics on the heart, and the need for therapies that treat cancer while limiting cardiac side effects. Notably, SGK1 is in the rare category of kinases, whose inhibition may be of benefit both in heart disease and cancer.

Using computational design and *in silico* screening, we identified a novel class of inhibitors for SGK1 to use as pharmacological tools to test the hypothesis that SGK1 inhibition can decrease I_Na_, shorten action potential duration and thereby rescue phenotypes associated with prolonged repolarization in CMs. Our results demonstrate proof of concept for SGK1 inhibition as a therapeutic target for cardiac arrhythmias and identify a small molecule lead, that with further medicinal chemistry can form the basis for novel and specific SGK1 inhibitors.

## Results

### CADD approach successfully identified a novel class of SGK1 inhibitory compounds

Based on previous data that SGK1 regulates I_Na_^4^, we sought to identify small molecule SGK1 inhibitors for use as pharmacological probes to validate SGK1 as a therapeutic target in the treatment of cardiac arrhythmias. Using a combination of structure-based and ligand-based virtual screens (SBVS and LBVS) to query a reference library for small molecule inhibitors of SGK1, we clustered compounds based on previously validated metrics of activity (using a filtered library for Lipinski properties (rule-of-5)^21^). From a collection of ~500,000 small molecules meeting our initial activity requirements, we identified ~1000 compounds using requisite structure-function details extracted from other benchmarked kinase inhibitors.

**Figure 1:**
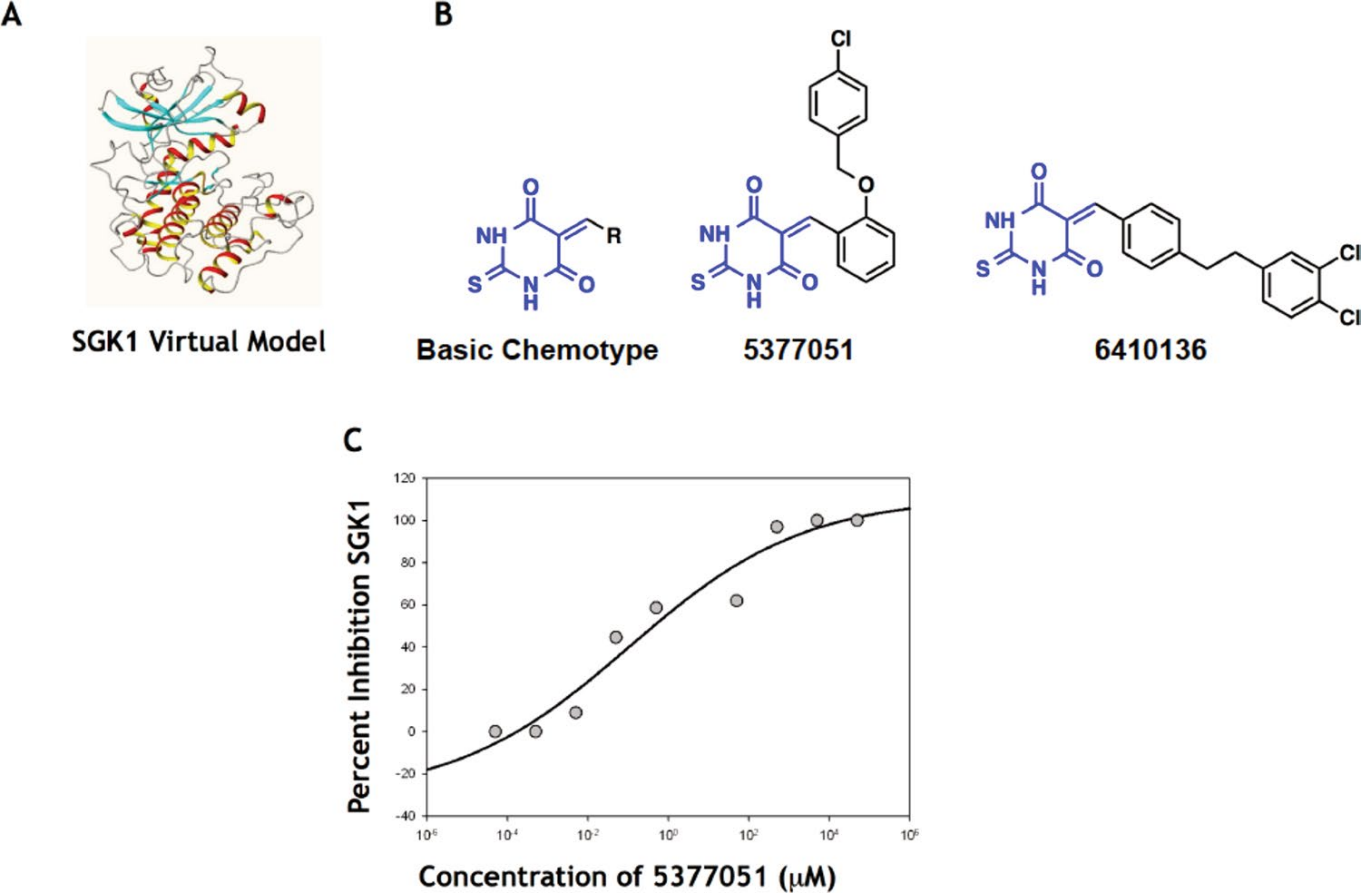
Computer-Aided Drug Discovery for SGK1 inhibitors. (**A**) Virtual model for SGK1 used for CADD. (**B**) SGK inhibitors were R-substituted-methylene-2-thioxodihydropyrimidine-4,6(1H,5H)-dione (left) derivatives with the two leading candidate structures shown (right). (**C**) Dose response curve for lead inhibitor 5377051 (in presence of 1 ng of recombinant SGK1). Dilutions of the inhibitor were added to recombinant SGK1 and SGK1 activity was assayed using a fluorescence polarization assay in triplicates.

In the absence of a high resolution structure of *active* SGK1 determined by X-ray crystallography or NMR spectroscopy at the time of the screening, we used comparative protein modeling to build a three-dimensional model for SGK1 based on known crystal structures of related proteins with acceptable sequence homology and protein folds. The model was later updated with the SGK-1 crystal structure (PDB code: 2R5T) and conformations of ‘activation loop’ sampled. This model (Fig. 1A) was used as the target for screening our initial 1st generation hits *in silico*. These small molecules were scored and ranked versus benchmarked kinase inhibitors that were doped into the library as reference compounds. These studies identified a final list of ~50 small molecules, representing 8 chemotypes that were then evaluated using an *in vitro* kinase assay to evaluate their ability to inhibit SGK1 kinase activity.

After validating the reproducibility and dynamic range of the *in vitro* kinase assay using recombinant GST-purified SGK1, we used this assay to evaluate the 1^st^ generation small molecule inhibitors identified using our CADD platform. Compounds with demonstrable SGK1 inhibitory potential were re-screened using an 8-point dosing curve to obtain IC50s. Several of the chemotypes had IC50s in the high nanomolar/low micromolar range, and one of these was chosen as a scaffold (R-substituted-methylene-2-thioxodihydropyrimidine-4,6 (1H,5H)-dione) for the second generation screen.

The results from our initial studies were then used to refine the true-positive (active) and false-positive (less active, decoy) ligand-based pharmacophore models in an iterative discovery process. The chemotypes identified using the LVBS in our second-generation screen were filtered as detailed in the methods section to exclude chemicals that do not dock within the ATP-binding site of the SGK1 model. This 2^nd^ generation screen yielded an additional 64 compounds that were again assessed using the *in vitro* kinase assay to exclude chemicals with poor SGK1 inhibitory activity. 9-point dose response curves on selected inhibitors that showed SGK1 inhibition at 20 μM dosage, identified several potential lead compounds (Fig. 1B). Based on a cell culture assay (see below), we focused on 5377051 and determined its IC_50_ (in cell culture) to be 2.1 μM (Fig. 1C).

### SGK1 inhibitor 5377051 targets SGK1 activity in primary cultured CMs

**Figure 2:**
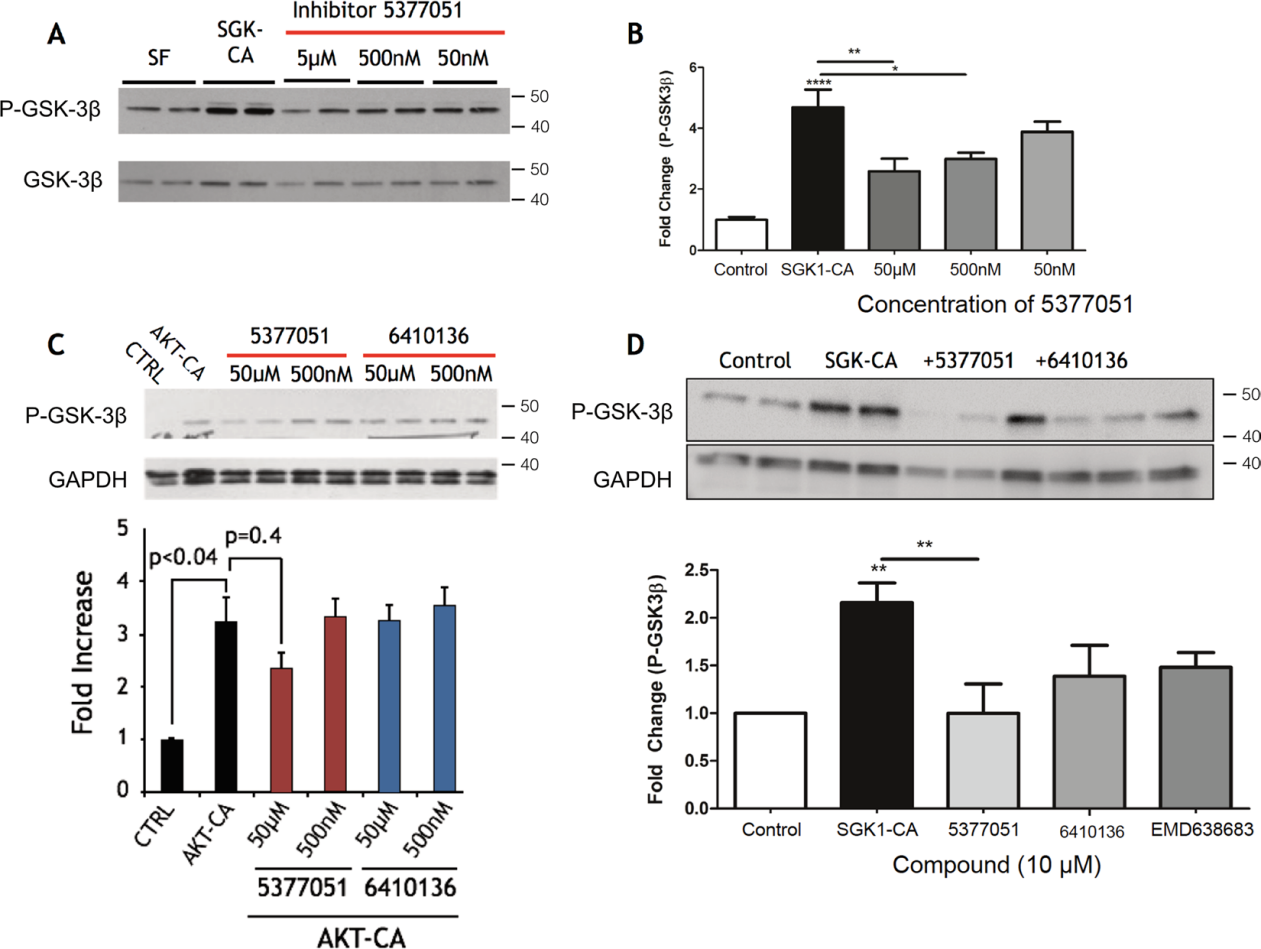
Inhibition of SGK1 in cultured cardiomyocytes by lead compounds. (**A**) Inhibition of SGK1 activity assessed by GSK3β phosphorylation in CMs infected by Ad.SGK1-CA. (**B**) Different concentrations of 5377051 were assessed at 48 hours after treatment with immunoblotting (left) and quantitated (right). (**C**). Effect of SGK1 inhibitors on Akt-induced phosphorylation of GSK3-beta in CMs infected by Ad.-myr-Akt was assessed by immunoblotting. (**D**) Comparison of potency of 10 micromolar compounds 5377051, 6410136 with the published inhibitor EMD63863. Immunoblots were cut to facilitate the incubation the same samples with multiple antibodies and cropped for clarity. All samples were loaded contiguously as shown. Quantified data are the composite of three independent experiments, each with three technical replicates. Each experimental set was conducted with the same batch of adenovirus.

As a first measure of the biological effectiveness of our putative SGK1 inhibitors in living cells, we examined the phosphorylation of an SGK1 target in cultured neonatal rat ventricular myocytes (NRVMs)^22^. Cells were infected with either an adenovirus expressing a constitutively active form of SGK1 (Ad.SGK1-CA) or a control adenovirus (Ad.GFP). At the time of infection, cells were also treated with various concentrations of SGK1 inhibitors 5377051 and 6410136, two of the molecules identified from the second-generation CADD-generated screen and validated as SGK1 inhibitors in the *in vitro* kinase assay. Cell lysates were subjected to immunoblotting with an antibody against phospho (ser38)-glycogen synthase kinase beta (p-GSK3β), a well-established SGK1 substrate 48 hours after adenoviral infection. As expected, Ad.SGK1-CA caused an increase in p-GSK3β, which was inhibited by both 5377051 and 6410136 at sub-micromolar concentrations, suggesting inhibition of SGK1 activity in cardiomyocytes (Fig. 2A,B, Supplementary Fig. 1).

Because SGK1 and Akt are structurally related but have different roles in the heart^23^, we next wanted to determine whether our putative SGK1 inhibitors would affect Akt signaling. NRVMs were infected with an adenovirus expressing constitutively active Akt1^24^ (Ad.myr-Akt) or a control adenovirus, and treated with the inhibitors as above. We again assessed levels of p-GSK3β, which is a common substrate for Akt and SGK1. While infection of NRVMs with Ad.myr-Akt caused the expected increase in phosphorylated GSK3β, incubation with either 5377051 or 6410136 failed to significantly inhibit Akt activity even at concentrations of 50 μM (Fig. 2C).

Finally, we directly compared the activity of our putative SGK1 inhibitors 5377051 and 6410136, to an SGK1 inhibitor, EMD 638683, previously reported by Merck Serono^25^. In a similar experimental design, our lead compounds showed equivalent SGK1 inhibition compared to the commercially available EMD 638683 (Fig. 2D).

### SGK1 inhibitor 5377051 blocks the SGK1-mediated phosphorylation of NDRG1 and proliferation of a prostate cancer cell line

**Figure 3:**
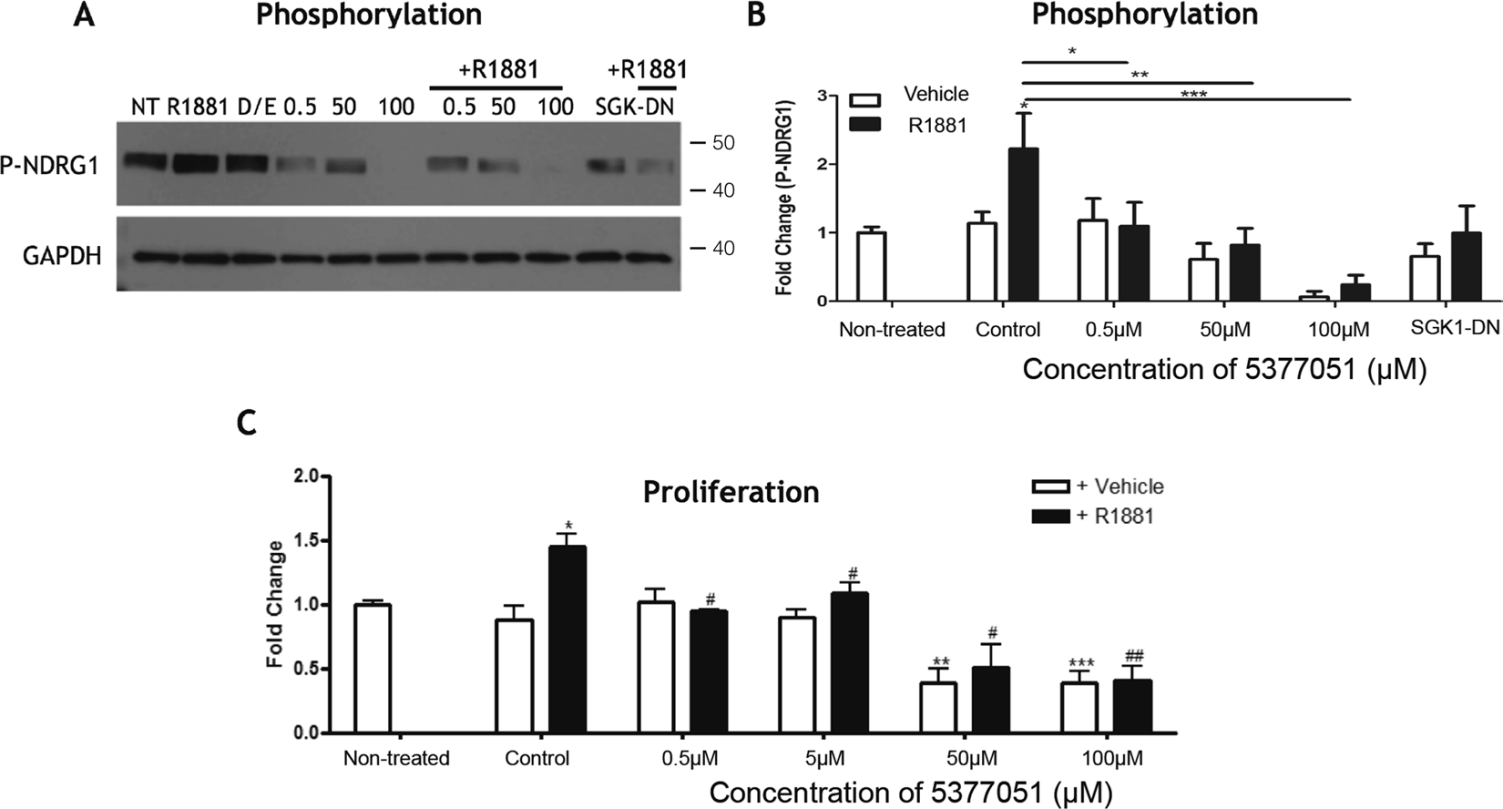
Validation of lead SGK1 inhibitor compound in a prostate cancer cell line. (**A**) Western blot demonstrating inhibition of SGK1 by compound 5377051 blocked the phosphorylation of SGK1 target NDRG-1 in response to the androgen analog R1881. (**B**) Quantification of NDRG-1 phorsphorylation. (**C**) 5377051 inhibited the proliferation of LNCaP prostate cancer cell line induced by the androgen analog R1881 as assayed by Cy-quant assay. Immunoblots were cut to facilitate the incubation the same samples with multiple antibodies and cropped for clarity. All samples were loaded contiguously as shown.

SGK1 is known to be up-regulated after androgen receptor activation in prostate cancer and plays an important role in androgen receptor dependent tumor proliferation and survival^26^. Accordingly, SGK1 inhibitors have been proposed as potential therapeutic approaches for prostate cancer^27^. We used the prostate cancer cell line, LNCaP, as a second independent assay for validation of the specificity and efficacy of our SGK1 inhibitors in a cell culture system, as has been previously reported by other investigators^14^. Cell lysates for LNCaP cells stimulated with the synthetic androgen R1881 with or without pre-incubation of compound 5377051, were assessed by immunoblotting for phosphorylation of the specific SGK1 target protein N-myc Downstream Regulated 1 (NDRG1), which is involved in stress responses and cell growth^28^. As expected, addition of R1881 led to a significant increase in phosphorylated NDRG1 (1.7 ± 0.2 fold p < 0.05). LNCaP cells transfected with SGK1-DN plasmid showed no increase in phosphorylation of NRDG1 after R1881, confirming that this effect is SGK1-dependent. The R1881-mediated increase in NRDG1 phosphorylation was also abrogated by treatment with 5377051 at concentrations as low as 500 nM (Fig. 3A,B).

We next assessed whether the SGK1 inhibitor would affect androgen-dependent cell proliferation in the LNCaP cell line. Addition of R1881 led to a significant increase in cell proliferation in LNCaP cells (1.5 ± 0.1 fold, p < 0.05), which was inhibited with increasing concentrations of compound 5377051 (1.093 ± 0.08 p < 0.05, 0.51 ± 0.18 p < 0.0001, 0.40 ± 0.1 p < 0.0001 fold respectively for the 500 nM, 5 μM and 50 μM doses compared with unstimulated cells, (Fig. 3C). Baseline cell proliferation was also inhibited at 50 μM and 100 μM although at these higher concentrations, we cannot exclude an effect of SGK1 inhibition on cell survival in addition to proliferation. Thus the lead inhibitor identified blocks SGK1 activity in the LNCaP prostate cancer cell line in addition to primary CMs, thereby providing additional validity of SGK1 as a target of 5377051.

### SGK1 activity regulates Na_V_1.5-mediated sodium flux

We had previously shown that transgenic mice with chronic activation of SGK1 in the heart had an increased propensity to ventricular arrhythmias, and that this was likely due to a marked alteration in I_Na_^4^. As a prelude to determining the effectiveness of SGK1 inhibition to treat ventricular arrhythmias, we first tested the hypothesis that SGK1 directly regulates the cardiac voltage-gated sodium channel (Nav 1.5) activity in a heterologous expression system.

HEK 293 cells stably expressing the SCN5a gene^29^, encoding Na_V_1.5, (referred to as HEK-Na_V_1.5) were transiently transfected with a plasmid containing either a constitutively-active form of SGK1 (SGK1 S422D, referred to as SGK1-CA), a dominant negative form of SGK1 (SGK1 K127M, referred to as SGK1-DN) or a vector expressing GFP only. Cells were studied by whole-cell voltage clamp 24–48 hours after transfection.

**Figure 4:**
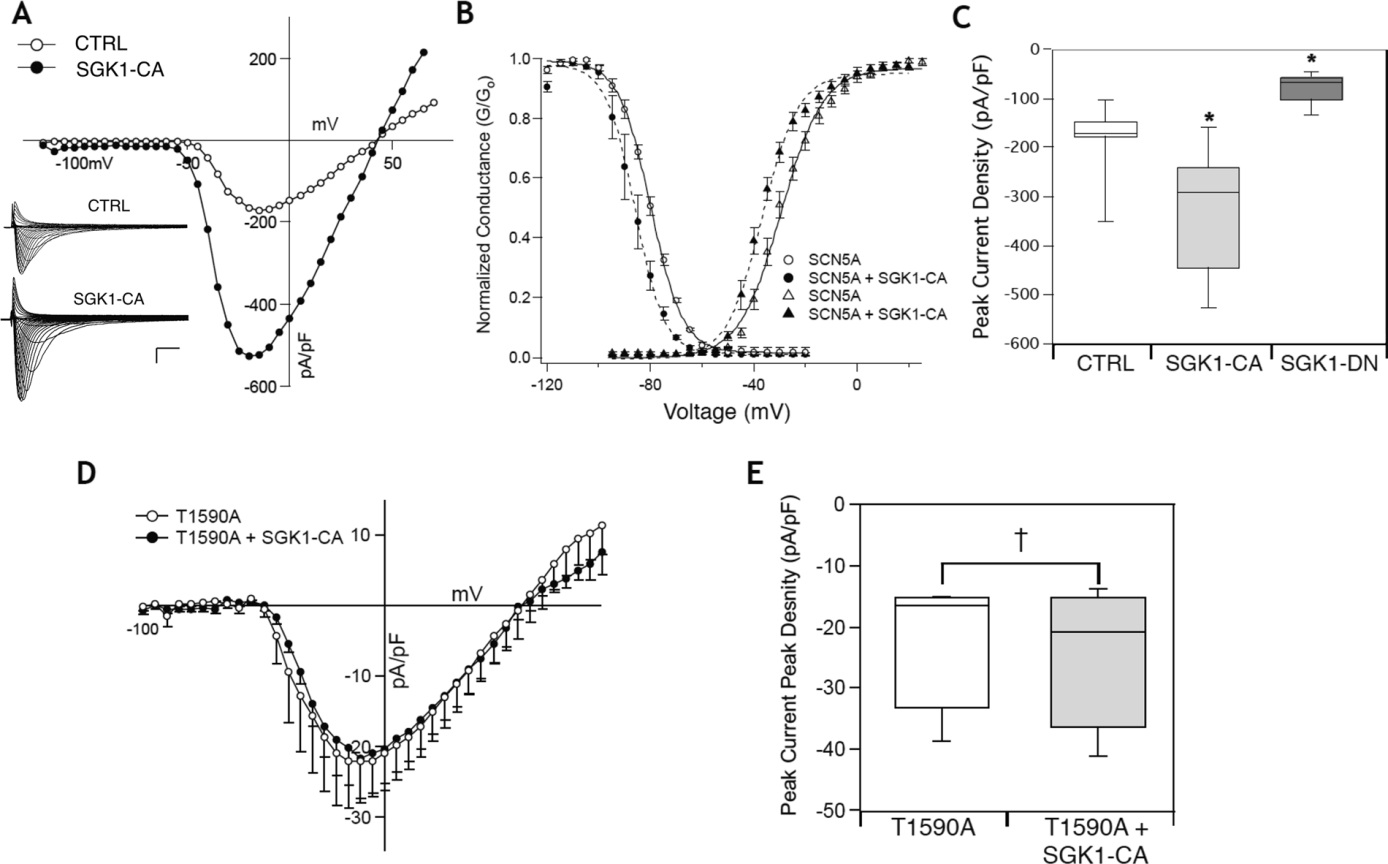
SGK1 regulates Na_V_1.5 channel function. (**A**) Expression of SGK1-CA in HEK-Na_V_1.5 cells leads to increase in I_Na_ density. Inset scale = vertical: 50 pA/pF, horizontal: 2 ms. (**B**) SGK1 activation leads to a hyperpolarizing shift of steady state activation and inactivation curves for I_Na_. (**C**) Peak sodium currents are increased with expression of SGK1-CA while inhibition of SGK1 by the dominant negative kinase dead SGK-1 (SGK1-DN) leads to decrease in I_Na_ density (*p-value < 0.005 by one way ANOVA, n = 5–11 for each condition). (**D**) Mutation of a novel SGK1 putative site T1590A leads to abrogation of the increase in I_Na_ with SGK1 activation. (**E**) Quantification of peak sodium currents (NS = non-significant change, n = 3–5 for each condition).

Expression of SGK1-CA led to a significant increase in I_Na_, while expression of SGK1-DN led to a decrease in sodium current density (Fig. 4A,C) compared to GFP controls. This was in agreement with our previous data, where constitutive activation of SGK1 in the heart led to an increase in I_Na_ in adult CMs isolated from the transgenic mice hearts^4^. In addition, HEK-Na_V_1.5 cells transfected with SGK1-CA had a leftward (hyperpolarizing) shift in steady state activation and inactivation (Fig. 4B), again recapitulating effects seen in CMs from SGK1-CA transgenic mice. This is also expected to increase the ‘window’ current, leading to an effective increase in I_Na_. Conversely, the inhibition of SGK1 in cells transfected with SGK1-DN led to a decrease in I_Na_ current density suggesting substantial baseline SGK1 activity in the HEK cells (unlike in CMs) that drives sodium flux in these cells (Fig. 4C).

We had previously identified three candidate target sites for SGK1 phosphorylation on Na_V_1.5, including two sites in the linker between subdomains I and II, and one in the C-terminal domain^4^. The first two sites had previously been shown by others to influence channel biophysical properties in oocyte expression experiments^30^, while the third site had not previously been identified. Mutation of these serines to alanines (to abolish SGK1 phosphorylation) also abolished the hyperpolarization shifts in the activation and inactivation curves described above (data not shown). Interestingly, mutation of the C-terminal residue threonine to alanine (T1590A), effectively preventing the phosphorylation by SGK1 at that site, dramatically reduced the SGK1-induced increase in sodium flux (Fig. 4D,E). Taken together, our data suggest that SGK1 regulates I_Na_ through multiple phosphorylation sites, and inhibition of SGK1 would be expected to reduce I_Na_ in pathological conditions.

### SGK1 inhibitor 5377051 reverses the SGK1-induced changes in I_Na_

**Figure 5:**
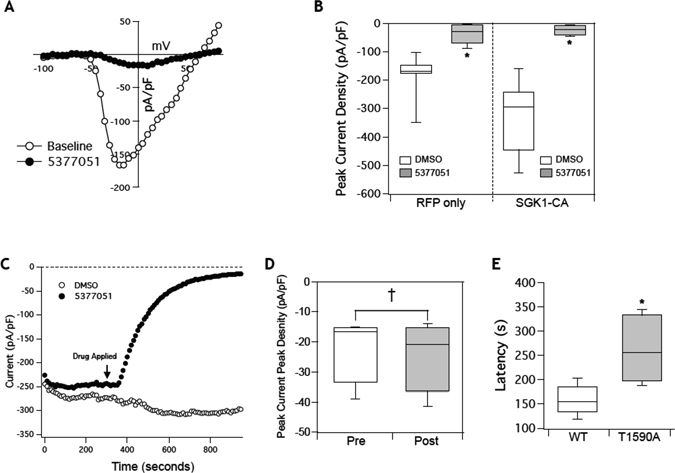
Inhibition with lead compound 5377051 modulates I_Na_ by inhibiting SGK1. (**A**) Addition of 5377051 at 10 uM significantly decreases I_Na_ even with co-expression of SGK1-CA. (**B**) Quantification of changes in peak sodium current with addition of inhibitor 2 (5377051) with and without co-expression of SGK1-CA (n = 4–6 per condition, *p-value < 0.05). (**C**) Representative time course of inhibition by compound 5377051 as compared to addition of DMSO. (**D**) Average time for inhibition demonstrating a half-time of 200 s (n = 4).

Because we had previously found that SGK1 regulates I_Na_, we sought to determine if pharmacological SGK1 inhibition could modulate I_Na_ in the HEK-Na_V_1.5 cells. Bath application of compound 5377051 at 10 μM resulted in a significant reduction in I_Na_ (Fig. 5A,B). Quantification of peak I_Na_ with and without co-expression of SGK1-CA in the presence of the inhibitor demonstrated an 80–90% reduction in sodium current, SGK1-CA (Baseline: 251.6 ± 62.2 pA/pF, +Inhibitor: 23 ± 9.8 pA/pF, n = 4, p < 0.05, Fig. 4B), RFP only (Baseline: 161.5 ± 32.0 pA/pF, +Inhibitor: 36.2 ± 17.4 pA/pF, n = 5, p < 0.005, Fig. 5B). The level of reduction of I_Na_ was similar to what was noted with genetic SGK1 inhibition by SGK1-DN expression. The inhibition by compound 5377051 occurs over approximately 6 minutes with a half-time of 158 ± 31.2 s (n = 6) (Fig. 5C).

To confirm that compound 5377051 was not a direct channel blocker we expressed the phosphorylation mutant T1590A in HEK cells and measured the peak I_Na_. There was no significant reduction in peak current of cells expressing the T1590A mutant channels when measured at a time point where there was more than 70% inhibition of wild-type Na_V_1.5 (Fig. 5D). While there was a reduction of I_Na_ in some cells expressing the T1590A mutation, the latency of inhibition was significantly prolonged (Fig. 5E). Together these data suggest that the effect of the SGK1 inhibitor on I_Na_ was critically dependent on SGK1 phosphorylation of Nav1.5, and that abolition of the phosphorylation sites on Nav1.5 also abolished the effect of 5377051. Hence our results supported our hypothesis that the action of compound 5377051 on I_Na_ current density was mediated by SGK1 inhibition and not an ‘off-target’ effect as a direct channel blocker.

### SGK1 inhibition in mammalian cardiomyocytes selectively reduces the late sodium current I_NaL_

Multiple lines of evidence have demonstrated that the late sodium current I_NaL_ is unregulated in pathological settings^31^. Mutations that lead to congenital long QT syndrome type 3 often increase the persistent sodium current and predispose cells to early after depolarizations (EADs) and ventricular arrhythmias^32^. Importantly, the increased arrhythmogenesis in the transgenic mice with SGK1 activation in the heart, could be attributed to an increase in I_NaL_^4^. However, the HEK-Na_V_1.5 cells do not demonstrate appreciable I_NaL_, likely due to the fact that the full voltage-gated sodium channel macro-molecular complex is not effectively reconstituted in this heterologous expression system. We therefore assessed the effect of pharmacological inhibition of SGK1 on native I_NaL_ in cultured primary cardiomyocytes (NRVMs).

**Figure 6:**
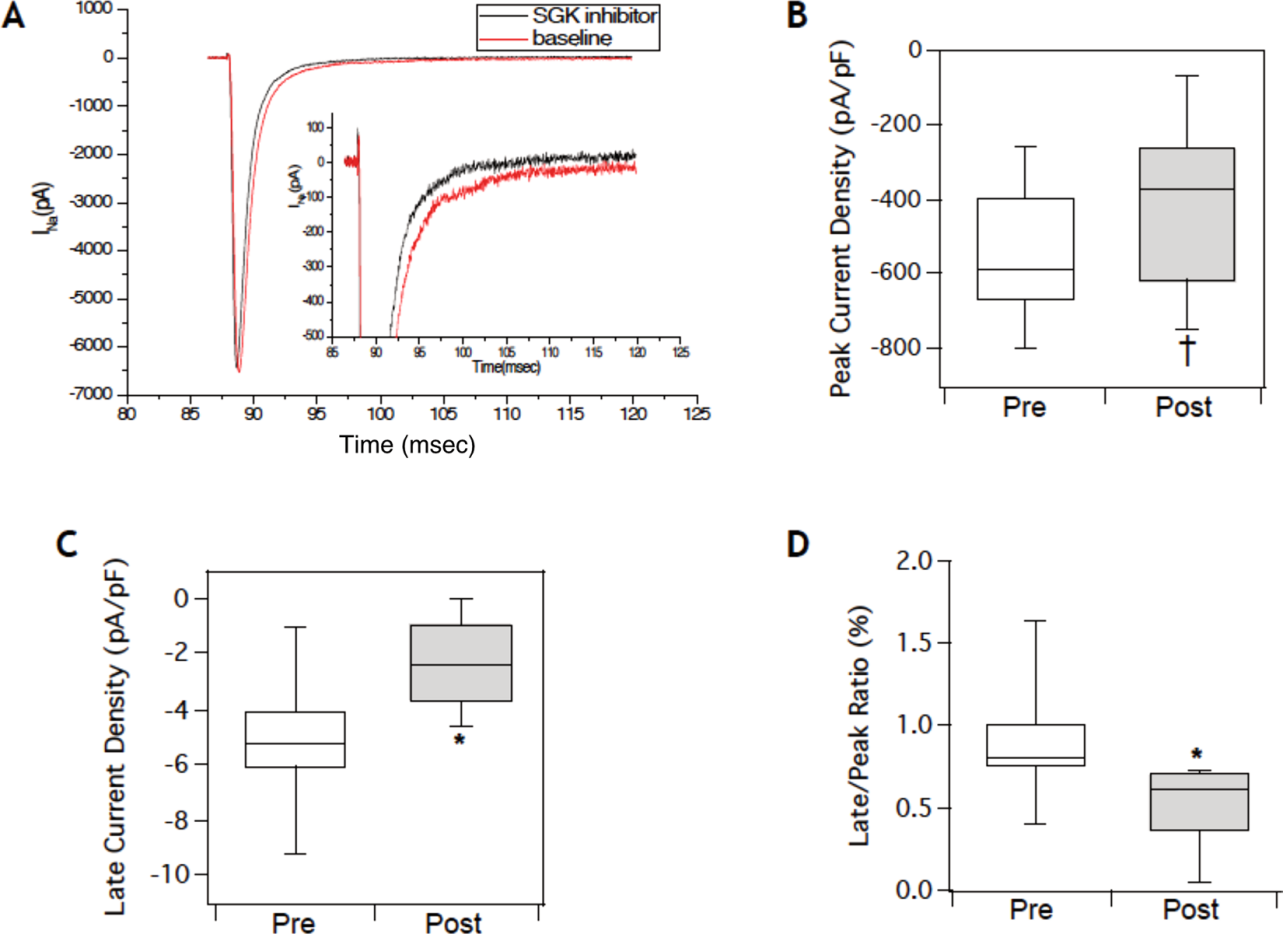
Inhibition of SGK1 in cardiomyocytes selectively reduces the late sodium current I_NaL_. (**A**) Representative I_Na_ from NRVMs at baseline and after acute treatment with compound 5377051 demonstrates preferential reduction of late current. (**B**) Quantification of the peak current does not demonstrate a significant reduction with administration of the inhibitor (Peak current density: Pre = −560.1 ± 180 pA/pF versus Post = −396.1 ± 230.4 pA/pF). (**C**) Selective inhibition of native late sodium current with incubation of the lead compound (Late current density: Pre = 5.12 ± 2.4 pA/pF versus Post = −2.3 ± 1.5 pA/pF). (**D**) Relative decrease in peak to late current ratio. (*p-value < 0.05, ^†^p-value = non-significant, n = 6 for all experiments).

Whole-cell recordings demonstrated a characteristic rapidly activating and inactivating inward sodium current to a single depolarizing pulse (Fig. 6A). Incubation with our lead compound for at least 5 minutes, did not result in a significant decrease in peak I_Na_ (Fig. 6B). While expectedly, the baseline I_NaL_ was small in native cardiomyocytes, there was a notable reduction in the measured late current after incubation with the compound (Fig. 6C), which appeared selective over the peak current (Fig. 6D). These data suggest that inhibition of SGK1 in native cardiomyocytes preferentially inhibits the late sodium current and may be a potential therapeutic target for forms of inherited heart disease such as long QT syndrome.

### Inhibition of SGK1 rescues 2:1 AV block due to prolonged QT in the breakdance zebrafish mutants

**Figure 7:**
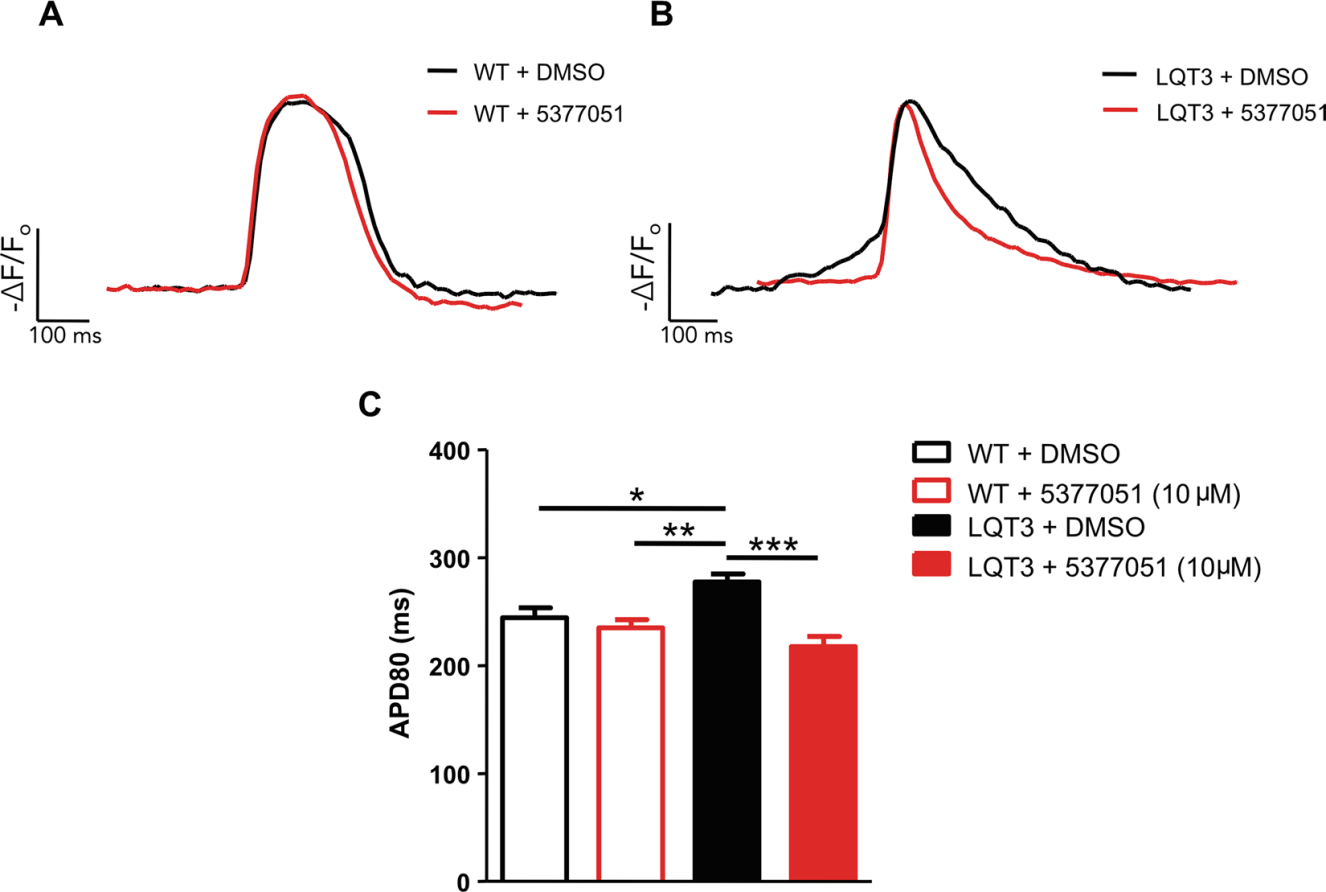
Treatment with SGK1 inhibitor 5377051 restores prolonged APD in a cellular model of LQT3. (**A**) Representative fluorescent tracings of spontaneous action-potentials from wild-type hiPSC-CMs expressing ArcLight with or without the SGK1 inhibitor 5377051 quantified by measuring the action potential duration at 80% repolarization (APD80) (WT + DMSO, 244 ± 9 ms, n = 15 versus WT + 5377051, 235 ± 8 ms, n = 11) (**B**) Fluorescent action potential recordings from LQT3 hiPSC-CMs with 5377051 demonstrate significant shorting of the APD80 (LQT3 + DMSO, 278 ± 7 ms, n = 22 versus LQT3 + 5377051, 218 ± 10 ms, n = 16). (**C**) Cumulative APD data from WT and LQT3 hiPSC-CMs with and without SGK1 inhibitor. Statistics was performed by applying a one-way ANOVA with Bonferroni’s post hoc correction: *P < 0.05, **p < 0.01 and ***P < 0.001, versus LQT3 + DMSO.

The zebrafish *breakdance* mutant (*bkd* ^−/−^) fish is a unique model of action potential prolongation due to a mutation in the zebrafish homologue of the KCNH2 channel recapitulating human LQT2 syndrome^33^. The phenotypic manifestation of the prolonged QT interval in the *bkd* ^−/−^ fish is the presence of functional 2:1 atrioventricular (AV) block (as is sometimes seen at in human neonates with LQTS as a consequence of prolonged repolarization and effective refractory period^34^), and previous high throughput experiments have taken advantage of this easily quantifiable phenotype to screen for compounds that shorten the action potential duration (APD) and ‘rescue’ the 2:1 AV block (Fig. 7A)^35^. This type of compensatory QT shortening has recently been demonstrated as a potential therapy in congenital long QT syndrome by inhibiting I_Na_ in a patient with Timothy syndrome^36^. *Bkd* ^−/−^ fish represent an animal model of long QT syndrome that provides a unique system to rapidly test the efficacy and potential toxicity of bio-active compounds in an animal model^37^.

To determine if SGK1 inhibition would also shorten the APD in the *bkd* ^−/−^ mutants, fish were injected with 500 μM of SGK1 morpholino or SGK1-DN mRNA (that inhibits SGK1 activity in a dominant negative fashion) at the 1–2 cell stage and scored for the presence or absence of 2:1 block at 99 hours post fertilization. The SGK-1 morpholino rescued 58% of mutants, compared with 5% spontaneous reversion in control-scrambled morpholino injections (Fig. 7B). Expression of SGK1-DN was even more potent, resulting in rescuing 90% of mutant fish (Fig. 7B). Thus two independent genetic approaches to SGK1 inhibition rescue the 2:1 AV block phenotype in this zebrafish model of LQTS. While the mechanism of QT shortening and rescue in this model by genetic SGK1 inhibition was not clearly evident, we exploited this finding to test the efficacy of our SGK1 inhibitors.

Pre-incubation of zebrafish homozygous for the *breakdance* mutation (Bkd^−/−^) for 24 hours with compound 5377051 resulted in effective rescue of 2:1 AV block in a dose-dependent fashion (Fig. 7C,D). The percent rescue with 45 μM of the inhibitor was similar to that seen with administration of a morpholino against SGK1. Minimal toxicity was seen at even higher concentrations of the inhibitor and did not appear to be dose-dependent (Fig. 7C). These data suggested that SGK1 inhibition by 5377051 may be potentially therapeutic for arrhythmic conditions associated with a prolonged QT interval.

### SGK1 inhibitor 5377051 shortens APD in human induced pluripotent stem cell (iPSC)-derived cardiomyocytes from a patient with LQT3

**Figure 8:**
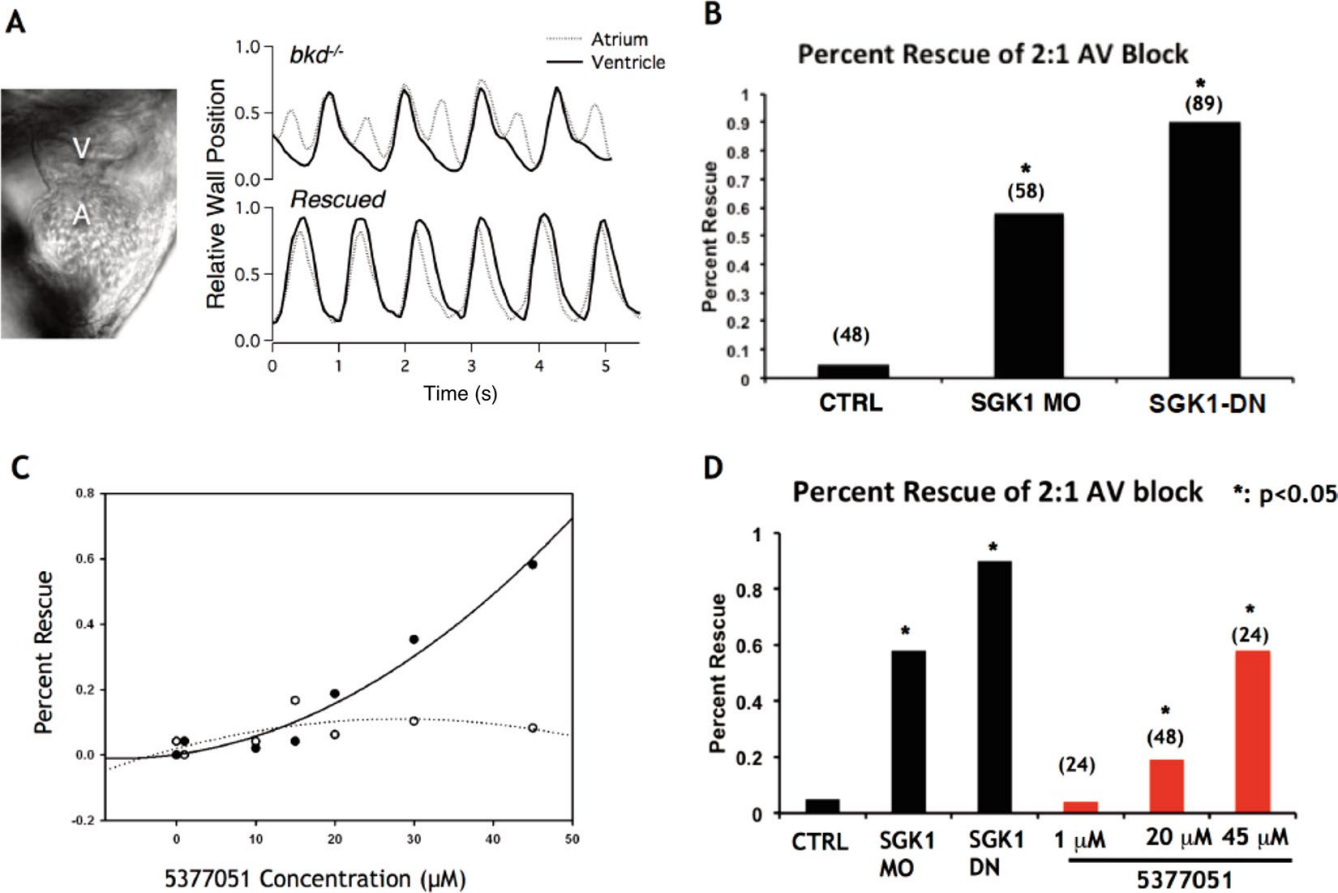
SGK1 inhibition rescues a zebrafish model of long QT. (**A**) Still frame image of zebrafish heart with chamber labels (A = atrium) (V = ventricle). Representative tracings of chamber wall position demonstrating 2:1 AV block in mutant *bkd* ^−/−^ zebrafish (upper panel) and with rescue of the phenotype (lower panel). (**B**) Knock-down with SGK1 morpholino or inhibition of SGK1 (500 μM SGK1-DN mRNA) in the *breakdance* zebrafish mutants with long QT at 1–2 cell stage leads to significant rescue of 2:1 AV block at 99 hpf stage (5% spontaneous reversion at this stage); *p-value < 0.05 by Z-test. (**C**) Compound 5377051 added at 48 hours post fertilization (hpf) and assessed at 72 hpf for rescue of phenotype 2 with superimposed rescue (solid line) and toxicity (dashed lines) curves shown on left (**C**) and representative bar graph on right (**D**). *p < 0.05 by Z-test.

To examine the therapeutic effects of SGK1 inhibition in a relevant disease model, we tested our lead compound in cardiomyocytes differentiated from human induced pluripotent stem cells (hiPSCs) that offer a unique model to study inherited arrhythmia disorders^38^. Application of the SGK1 inhibitor did not significant prolong the action potential duration (APD) of hiPSC-derived cardiomyocytes (hiPSC-CMs) from a normal patient as measured by the fluorescent voltage sensor Arclight^39^ (Fig. 8A). In contrast incubation of our lead compound with hiPSC-CMs derived from a patient with a sodium channel defect causing LQT3 (Nav1.5-N406K)^40^, results in significant shortening of the APD and correction of the abnormal phenotype (Fig. 8B,C). These data suggest that SGK1 inhibition with our lead compound 5377051 can reverse the defect seen in LQT3 at a cellular level in a model relevant to human disease.

## Discussion

Alteration of I_Na_ has been implicated in both inherited and acquired arrhythmia syndromes, and plays a causative role in the etiology of some ventricular arrhythmias. Here we show that SGK1 is an important regulator of I_Na_, and that SGK1 inhibition represents a therapeutic strategy for the treatment of certain cardiac arrhythmias.

SGK1 inhibitors have previously been developed in the context of treatment for prostate cancer and one of these inhibitors has also been tested *in vivo* as an anti-hypertensive^25^. However, SGK1 inhibition as a novel therapeutic approach to modulating I_Na_ has not been previously explored to our knowledge. Using rational drug design along with directed screening, we have identified a novel scaffold for SGK1 inhibition that retains specificity for SGK1 over the closely related kinase Akt1. The compound 5377051 appears to inhibit SGK1 effectively and specifically in two different cell types, making it unlikely that our results are due to off-target effects. We anticipate that derivatives of this compound or other SGK1 small molecule inhibitors may have potential not only as a treatment for arrhythmias in inherited and acquired cardiac disease with abnormalities in I_Na_ flux, but may also protect against adverse cardiac remodeling as has been seen with genetic SGK1 inhibition in the heart^4^. Finally, as most kinase inhibitors used in oncological diseases have adverse cardiovascular effects, SGK1 inhibitors have the unique property of being potentially beneficial in both classes of disease.

While the potency of 5377051 *in vitro* was less than the potency of other kinase inhibitors, the IC50 in cell-based assays was as little as 1–5 μM with a minimal effect on the related kinase *Akt* at the same concentrations. While additional refinement through medicinal chemistry will undoubtedly be necessary (in addition to testing against other kinases to gauge off-target kinase inhibition), our lead compound represents a useful tool to validate SGK1 as a relevant target for sodium channel modulation.

In this study, our single cell electrophysiology experiments demonstrated the potency, biophysical effects and kinetics of the SGK1 inhibition on Na_V_1.5 channel activity, recapitulating many of the effects of persistently active SGK1 on Na_V_1.5 we had previously reported. These effects were further replicated by acute application of the lead compound 5377051 during whole cell patch-clamp recordings. The time course of inhibition was on the order of several minutes, which would be slow for channel inhibition by a typical pore blocker, but consistent with a post-translational mechanism. In addition, mutation of the putative SGK1 phosphorylation T1590 to alanine significantly altered the sensitivity of the channel to SGK1 inhibition by 5377051, further demonstrating that the effect is not due to direct channel blockade. In contrast to CMs, the dramatic degree of inhibition of I_Na_ in HEK cells by either genetic or pharmacological inhibition of SGK1 is likely secondary to a high level of activity of SGK1 in HEK cells because of their epithelial lineage.

Unlike our prior studies^4^, we did not observe the increase in late sodium current in our heterologous expression system in comparison to isolated cardiomyocytes from transgenic mice expressing constitutively active SGK1, likely due to the absence of the associated subunits such as the beta-subunits which can significantly modulate late current levels in HEK cells^41^ or related to reductions in external sodium necessary to fully clamp the peak current. However, in primary cardiomyocytes, our lead compound selectively inhibited the late sodium current over the peak current. Taken together our data strongly suggest that SGK1 is a potent modulator of the sodium channel and pharmacologic inhibition of SGK1 activity inhibits the late sodium current that is often associated with pathologic conditions^4^. Our studies provide ‘proof-of-concept’ of SGK1 inhibition as a therapeutic target for treating cardiac arrhythmias.

Extensive research over the last two decades has demonstrated that ion channels are complex macromolecular machines and parts of signaling networks with a diverse array of associated proteins. The cardiac sodium channel Na_V_1.5 is modulated by multiple post-translational modifications including phosphorylation^42^ and acetylation^43^. These modifications can directly affect the biophysical properties of the channel, change the number of available protein complexes at the plasma membrane or modulate associated proteins that can further affect channel function. While traditional ion channel blockers have efficacy in treating certain types of arrhythmias, modulation of ion channel post-translational modification may provide more fine-tuned therapy without the pro-arrhythmic side effects commonly seen in typical ion channel blockers. In support of this, there appears to be a ‘plateau’ effect for SGK1 inhibition *in vivo*, both in mice with chronic SGK1 inhibition in cardiomyocytes (which do not have shorter repolarization^4^), and in human iPSC-derived cardiomyocytes from normal patients (Fig. 7A). Furthermore, inhibition of hERG, a major concern for anti-arrhythmic therapies is not seen with the SGK1 inhibitor 5377051. Assessment of effect of 5377051 in HEK cells transfected with hERG, demonstrated an increase in tail currents of hERG in HEK cells treated with our lead compound compared to control (Supplemental Fig. 2), although this enhancement of hERG tail currents in HEK cells do not appear to cause any meaningful shortening of the action potential in iPSC-cardiomyocytes from a normal patient.as shown in Fig. 8A. While future derivative compounds will have to be thoroughly tested for off-target activity against other ion channels our preliminary results clearly rule out inhibition of the hERG channel by this class of compound while demonstrating a possible positive effect on hERG tail currents.

Finally, to test the feasibility of the compound as a functional cardiovascular drug, we demonstrated effective treatment of the zebrafish *breakdance* model of Long QT syndrome with minimal toxicity. The mechanism of rescue is likely secondary to an inhibition of inward sodium current that compensates for the impaired repolarization from reduced potassium flux, although this hypothesis was not verified. Alternatively, the rescue may be in part due to direct enhancement of hERG channel activity (Supplemental Fig. 2). While the exact mechanism for rescue of the breakdance phenotype was not elucidated, these data demonstrate the efficacy and low toxicity of the inhibitor in a more complex biologic system and suggest that SGK1 inhibitors could be used effectively in Long QT syndrome caused by a range of ion channel mutations.

Finally, to prove the validity of SGK1 inhibition in treating human arrhythmia disorders, we demonstrated the efficacy of SGK1 inhibitor in shortening the action potential duration in human iPSC-derived cardiomyocytes from a patient with known LQT3 due to a gain-of-function mutation in Na_V_1.5. These experiments provide clinical relevance of SGK1 as a target for treatment of ventricular arrhythmias in conditions with abnormal increases in sodium flux.

In conclusion, in this study, using CADD-based small molecule inhibitors of SGK1 as pharmacological probes to complement genetic inhibition of SGK1, we have demonstrated ‘proof-of-concept’ SGK1 inhibition as a therapeutic target for ventricular arrhythmias. While our lead compound represents a useful pre-clinical tool for target validation, future efforts at improving the potency and specificity of SGK1 inhibitors using medicinal chemistry will be needed prior to further testing in relevant pre-clinical models such as disease-specific iPSC-derived cardiomyocytes and animal models of long QT syndrome.

## Methods

### Computer Aided Drug Discovery (CADD) of SGK1 inhibitor chemotypes

All molecular modeling operations were performed in the SYBYL-X (Certara/Tripos) and/or BIOSOLVEIT (BioSolveIT, GmBH) molecular modeling packages, running on Dell Precision 690 workstations with 8 CPUs, 10 GB memory and RHE Linux 5 OS. The chemical libraries employed in the virtual screening primarily included Chembridge (www.Hit2Lead.com, San Diego, CA), pre-filtered for the Lipinski rule-of-five for drug-like properties. Ligand based virtual screen (LBVS) was performed using Sybyl, in which we used 2D and/or 3D conformations of known “ligands” including many of the identified kinase inhibitors (independent of target) on the basis of kinase inhibitory properties to construct multiple 3D pharmacophoric fingerprints to rapidly screen small molecule libraries *in silico.* The 3D-pharmacophore fingerprint/key we used is an extension of the 2D-pharmacophore models that are commonly used by others^44^. Briefly, these keys are comprised of functional groups responsible for mediating critical ligand receptor interactions and are represented as potential pharmacophore points (PPPs) in three-dimensional space. These PPPs are comprised of triplets or quartets with a triplet PPP using three pharmacophore points that include requisite distance data and/or quartets that are comprised of four pharmacophore points along with six distance and/or chirality constraints^45^. High-throughput docking (HTD)-based virtual screening was performed using FlexX and FlexX-ensemble, which consider the conformational flexibility within the target interface, in combination with a powerful incremental construction algorithm that allows the complexity and size of the ligand to be iteratively constructed from a predefined base fragment^46^. The algorithm used by FlexX is based on the model of molecular interactions defined by Bohm^47^ and Klebe and is divided into three segments: core or base selection, core placement, and incremental complex construction^48^. After docking, a variety of scoring functions are used to energetically assess each calculated pose, which take into account forces involved in this interaction as well as the affinity between the interacting atoms. Importantly, we performed cross docking; pose generation validation studies using additional HTD algorithms including: Surflex-Dock (TRIPOS) and/or Glide (Schrödinger) to ensure our hit list was not biased towards a single HTD algorithm/approach.

### SGK1 kinase assay

To screen CADD-generated SGK1 inhibitor candidates, we used a fluorescence polarization-based *in vitro* kinase assay (Upstate). Briefly, this assay utilizes a phosphorylated peptide that is labeled with a green fluorescent dye and increases its fluorescence polarization value upon binding with a phospho-specific antibody. We first validated the reproducibility and dynamic range of the assay using different concentrations of a recombinant GST-purified SGK1 and determined the EC50 of the recombinant kinase to be 1 ng. This assay was then used to evaluate the 1^st^ and 2^nd^ generation small molecule inhibitors identified using our CADD platform. Initial activity screening was done at a concentration of 50 μM to ensure we did not miss compounds with activity and several compounds had demonstrable SGK1 inhibitory potential. These inhibitors were re-screened using an 8 point dosing curve to obtain IC50s for several of the inhibitors, and used for the second round of CADD to generate an additional 64 compounds for further testing in this kinase assay.

### SGK1 inhibition in cardiomyocytes

All experiments and protocols including animal procedures for isolation of cardiomyocytes, were approved by the Institutional Biosafety Committees (IBCs) and institutional IACUCs at both Beth Israel Deaconess Medical Center and Massachusetts General Hospital in accordance with institutional and national guidelines. To investigate the activity of SGK1 in cardiomyocytes, 2 × 10^6^ neonatal rat ventricular myocytes (NRVMs) were plated on 60 mm plates. 24 h after plating, serum was removed and infected with SGK1-CA virus and treated with SGK1 inhibitors. Twelve hours after infection and treatment, the media was changed and cells were treated again. Twelve hours following the second treatment NRVMs were harvested and separated by gel electrophoresis.

### SGK1 inhibition in prostate cancer cell line LNCaP

For Western Blotting, 2 million LNCaP (ATTC) cells were plated on 60 mm dishes in RPMI-1640 containing 10% FBS and 1% pen-strep antibiotic. After 24 h plating cell media was replaced with RPMI-1640 containing 10% charcoal stripped fetal bovine serum to decrease androgen interference from the media. Cells were either transfected with SGK-DN or treated with drug (500 nM–100 μM and treated with the synthetic androgen analog R1881 (1 nM). Cells were treated at 0, 12 and 24 h and harvested 32 h after first treatment. Proteins were separated by gel electrophoresis using 50 μg of protein per well. P-NDRG1 (Cell Signaling) was normalized to GAPDH and data presented as fold change from control. For CyQUANT cell proliferation assay (Life Technologies), LNCaP cells were plated at 25,000 cells/well in a 96 well plate in plating media. 24 h after plating, media was changed to RPMI-1640 containing 10% charcoal stripped fetal bovine serum. Cells were treated with either 5377051, R1881 (1 nM) or both every 24 h for 72 h. Cell proliferation was measured using the manufacturer’s protocol.

### Cellular Electrophysiology

HEK-293 cells with stably-transfected SCN5a were voltage clamped using the whole-cell patch-clamp configuration as previously described^29^. To determine the effects of either constitutively (SGK1-CA) active or dominant negative (SGK1-DN) SGK1, respective plasmids were transfected for 4–6 hours with Lipofectamine 3000 (Life Technologies) and recordings were performed the following day. To improve the selection of transfected cells SGK1-CA was fused to mCherry by standard molecular biology techniques. All control cells were transfected with constructs expressing GFP or mCherry alone. All *I*_*Na*_ recordings (Axon Instruments) were performed at room temperature with patch pipettes of 1.5–2.5 MΩ tip resistance when filled with pipette solution containing (in mM): NaCl 5, CsCl 40, glutamate 80, CsOH 80, Mg-ATP 5, EGTA 5, HEPES 10, CaCl_2_ 1.5 (free [Ca2+] = 100 nM, pH 7.2 with CsOH, LJP = +5 mV). The bath solution contained (in mM) NaCl 50, MgCl_2_ 2, CaCl_2_ 1.2, NMDG 80, HEPES 10, Glucose 10 (pH 7.4). In all experiments, recordings started 5 min after establishment of the whole-cell mode to permit stabilization of current amplitude, voltage dependence and kinetics of gating. Standard voltage protocols were used for assessment of the voltage-dependence of activation/inactivation, recovery from inactivation. To determine the membrane potential for V1/2 and the slope factor k, steady state inactivation data were fit with a Boltzmann function of the form: I/Imax = {1 + exp[(V − V1/2)/k]} − 1. For late sodium channel recordings, NRVMs were isolated and cultured on laminin-coated glass coverslips. Whole-cell recordings were performed 5 minutes after break-in and the external solution was similar to those used in the recordings of HEK293 cells, 50 mmol NaCl but without NMDG. We performed at least five current recordings per cell with an interval of 30 seconds between recordings, without drug and after bath exchange with the inhibitor (50 μmol/L). Currents before and after drug administration were averaged (n = 7 cells).

### Maintenance and differentiation of iPSCs into Cardiomyocytes

Skin fibroblasts were obtained from a healthy volunteer and reprogrammed into pluripotent stem cells by standard methods. HiPSCs from a patient with LQT3 were previously described^40^. Human iPSCs were maintained in Essential 8 media and passaged every 3–5 days as single cells on Geltrex-coated plastic dishes (Invitrogen). Differentiation was performed as described elsewhere by sequential inhibition of GSK3 and the Wnt signaling pathways^49, 50^. Spontaneously beating CMs between days 20 and 30 were dissociated with 0.25% Trypsin-EDTA (GIBCO), re-plated on Geltrex-coated 96-well plates and maintained in RPMI 1640 medium with B27 complete supplement (Invitrogen) as described previously^50^.

### Optical Imaging of ArcLight Fluorescence in hiPSC-CMs

Dissociated hiPSC-CMs were transduced with lentivirus carrying pLenti-CMV-ArcLight for 72 hr^50^. 5 days after the onset of transduction, CMs were incubated with DMSO or 5377051 (10 μmol/L) for 24 hrs. Cells were then imaged with a Nikon Eclipse Ti-U inverted microscope, a Nikon CFI S Fluor 20X objective, an ORCA-Flash2.8 C11440-10C digital CMOS camera (Hamamatsu) and the MetaMorph Microscopy Automation and Image Analysis Software at 37 °C. ArcLight fluorescence was obtained with a FITC filter set and a SOLA SE light engine for illumination (Lumencor). Images were recorded at 1000 frames per second. ArcLight recordings were analyzed using a custom-written Matlab program. Action potential duration at 80% repolarization (APD80) was calculated to be the time interval between when signals reached 50% of the difference in fluorescence between the initiation and maximal height of an upstroke, and 80% of the return to diastolic baseline. The peak-to-peak interval was used to determine beating rate and all APD80 analyzed was corrected to reflect ADP80 at 1 Hz. Optical AP tracings are presented as −ΔF/F_0_, where F_0_ is the resting diastolic fluorescence.

## Electronic supplementary material

Supplemental Information
