# Supplementary material for: Inhibition of serum and glucocorticoid regulated kinase-1 as novel therapy for cardiac arrhythmia disorders
Source: Sci Rep. 2017 Mar 23;7:346. doi: 10.1038/s41598-017-00413-3 (PMC5428512; doi:10.1038/s41598-017-00413-3)
Supplement: Supplementary file 1 — Supplemental Information [file 41598_2017_413_MOESM1_ESM.pdf]

Supplementary Information:

Inhibition of serum and glucocorticoid regulated kinase-1 as novel therapy for cardiac arrhythmia disorders

Vassilios J. Bezzerides[1,2], Aifeng Zhang[1,5], Ling Xiao[5], Bridget Simonson[1,5], Santosh A. Khedkar[1,3], Shiro Baba[6], Filomena Ottaviano[1], Stacey Lynch[4], Katherine Hessler[1], Alan C. Rigby[1,4], David Milan[5], Saumya Das[1,5*] and Anthony Rosenzweig[1,5*].

1. Beth Israel Deaconess Medical Center, Boston, MA.
2. Boston Children's Hospital, Department of Cardiology, Boston, MA.
3. Current address: ChemBio Discovery Solutions, Lexington, MA.
4. Warp Drive Bio Inc., Cambridge, MA.
5. Massachusetts General Hospital, Boston, MA.
6. Graduate School of Medicine Kyoto University, Kyoto City, Japan.

*: These authors contributed equally to the work.

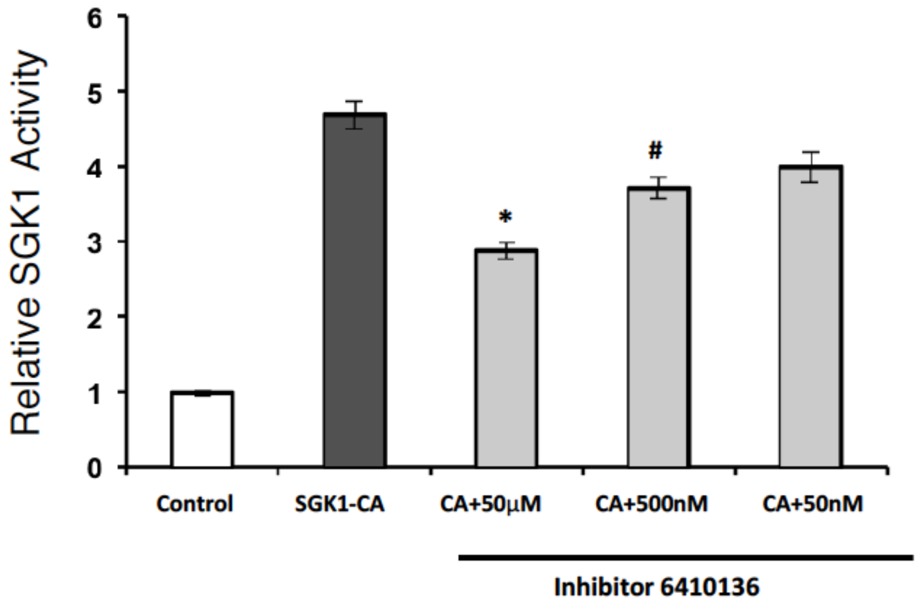

Supplemental Figure 1: Inhibition of SGK1 activity by additional SGK1 inhibitor compound 6410136 in cardiomyocytes

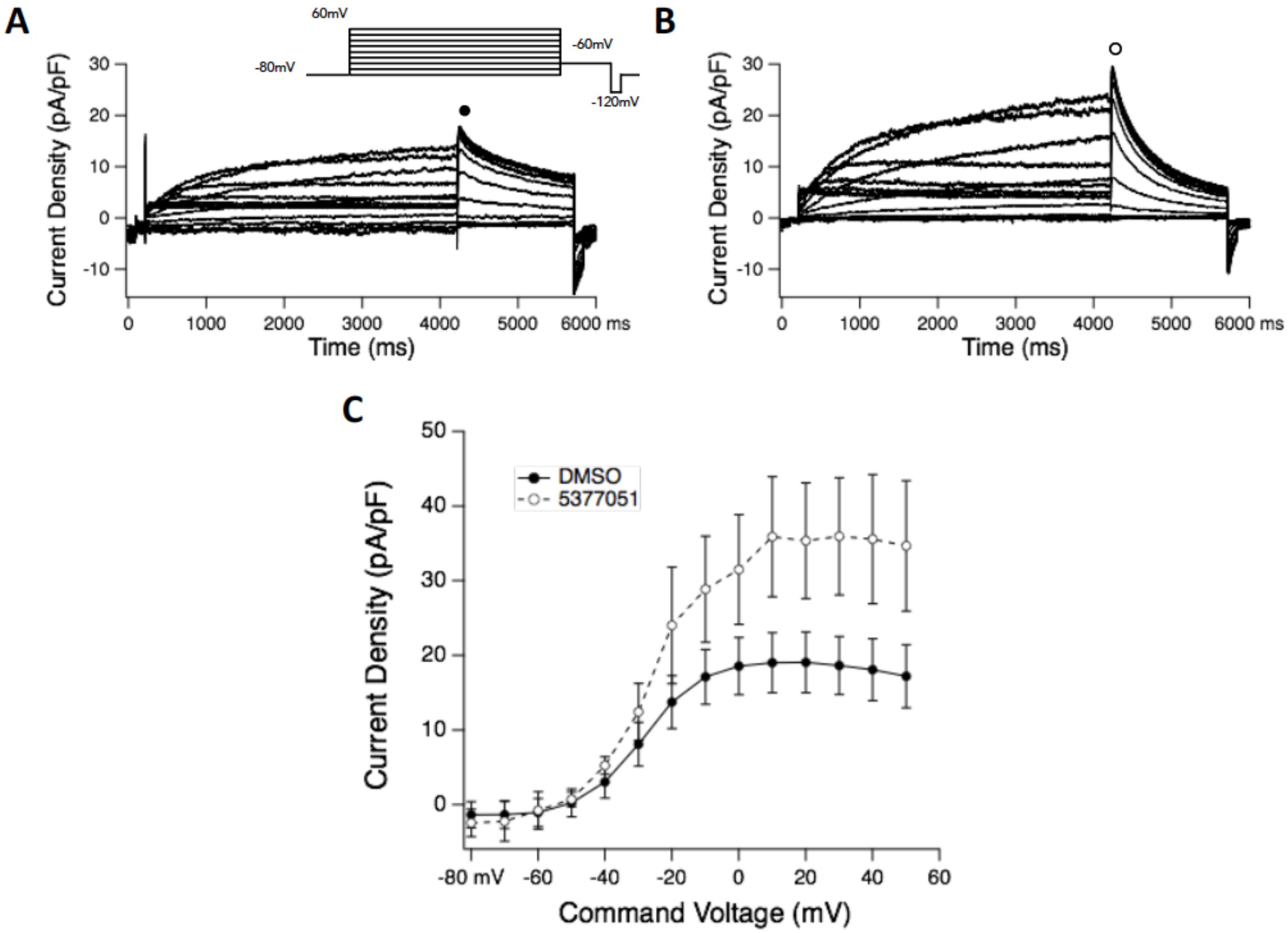

Supplemental Figure 2: Increase in hERG tail currents with SGK1 inhibitor. Representative traces from HEK cells expressing hERG either pre incubated for 5 minutes with **A**. DMSO or **B**. SGK1 inhibitor 5377051. **C**. Combined tail current data from n = 3-4 cells per condition, using voltage protocol as shown in inset in A and measured as indicated.
